# Estrogen-like activity of aqueous extract from *Agrimonia pilosa* Ledeb. in MCF-7 cells

**DOI:** 10.1186/1472-6882-12-260

**Published:** 2012-12-21

**Authors:** Young Min Lee, Jung Bong Kim, Ji Hyun Bae, Jong Suk Lee, Pan-Soo Kim, Hwan Hee Jang, Haeng Ran Kim

**Affiliations:** 1 Functional Food & Nutrition Division, Department of Agro-food Resources, National Academy of Agricultural Science, Rural Development Administration, Suwon, Republic of Korea; 2 Gyeonggi Biocenter, Gyeonggi Institute of Science and Technology Promotion, Suwon, Gyeonggi-do, Republic of Korea

**Keywords:** MCF-7 proliferation assay, Estrogenic activity, *Agrimonia pilosa*, Metabolic syndrome

## Abstract

**Background:**

Postmenopausal women experience estrogen deficiency-related menopausal symptoms (e.g., hot flashes and mood swings) and a dramatic increase in the incidence of chronic diseases. Although estrogen-replacement therapy (ERT) can reduce mortality from cardiovascular disease and improve osteoporosis and menopausal symptoms, its side effects have limited recent use. This study investigated the estrogen-like activity of aqueous extract from *Agrimonia pilosa* Ledeb.

**Methods:**

The estrogenic activity of *A. pilosa* was investigated by using several *in vitro* assays. The binding activity of *A. pilosa* on estrogen receptors was examined using a fluorescence polarization-based competitive binding assay. The proliferative activity of *A. pilosa* was also examined using MCF-7 cells. Furthermore, the effect of *A. pilosa* on the expression of 3 estrogen-dependent genes was assessed.

**Results:**

Using liquid chromatography-mass spectrometry, the 3 major peaks of *A. pilosa* aqueous extract were identified as apigenin-hexose, luteolin-glucuronide, and apigenin-glucuronide. The aqueous extract induced the proliferation of estrogen receptor-positive MCF-7 cells (*p* < 0.05). *A. pilosa*-stimulated proliferation was blocked on adding the estrogen antagonist ICI 182,780. Moreover, *A. pilosa* treatment increased the mRNA expression of the estrogen-responsive genes pS2 and PR (*p* < 0.05).

**Conclusions:**

These results suggest *A. pilosa* can be used to improve estrogen deficiency-related menopausal symptoms or to treat diseases in postmenopausal women.

## Background

Postmenopausal women experience estrogen deficiency-related menopausal symptoms, including hot flashes, mood swings, and sweating [1]. Postmenopausal women also exhibit a dramatic increase in the risk of metabolic syndrome, cognitive deficits, cardiovascular disease, dyslipidemia, and osteoporosis [2-5]. As the population ages and life expectancy increases, the importance of preventing and/or improving menopause-related changes has become paramount [6].

Hormone replacement therapy (HRT) can reduce mortality from cardiovascular disease and improve osteoporosis and menopausal symptoms [7]. In addition, Daly *et al.* reported that quality of life significantly improved after HRT [8]. However, many studies have shown that HRT is no longer a good solution for the treatment of menopausal women because of the increased risk of breast and endometrial cancers associated with long-term estrogen-replacement therapy (ERT) [9,10].

Phytoestrogens, that is, plant-derived estrogens, are found naturally in a diverse range of foods and include isoflavones, lignans, coumestans, and flavonoids (e.g., quercetin and kaempferol). Because the structure of phytoestrogens is similar to that of human estrogen, phytoestrogens can bind to estrogen receptors (ERs) [11]. Much scientific effort has been put into the search for additional phytoestrogens showing estrogen-like activity, leading to the continuous discovery of novel phytoestrogens in nature [12,13].

*Agrimonia pilosa* Ledeb. is a medicinal plant with anti-cancer [14], anti-oxidant [15], acetylcholinesterase inhibitory [16], and anti-inflammatory activities [17,18]. Some chemical studies have reported that *A. pilosa* contains phenolic compounds such as agrimonin, catechin, quercetin, and rutin [19,20]. However, the estrogenic effects of aqueous extract from *A. pilosa* have not yet been examined.

The purpose of this study was to investigate the estrogenic activity of *A. pilosa* by using several *in vitro* assays. The binding activity of *A. pilosa* on estrogen receptors was examined using a fluorescence polarization-based competitive binding assay. The proliferative activity of *A. pilosa* was also examined using MCF-7 cells. Furthermore, the effect of *A. pilosa* on the expression of 3 estrogen-dependent genes was assessed.

## Methods

### Plant material and extraction

The aerial parts of *A. pilosa* were purchased from Kyungdong Market (Seoul, Korea) in dried form and identified by the Classification and Identification Committee of the Korea Institute of Oriental Medicine (KIOM). A voucher specimen (KIOM109-122Aa) has been deposited at the herbarium of the Department of Herbal Resources Research of the KIOM. Each of the dried components was extracted twice with 10 volumes of water at 80°C for 3 h. The extracts were filtered through filter paper (Whatman, Maidstone, UK) and were concentrated under reduced pressure by a rotary evaporator (EYELA, Tokyo, Japan) at 40°C. The water filtrates were frozen and lyophilized. The lyophilized extracts were stored at −20°C until use.

### Analysis of *A. pilosa* aqueous extract composition

The chemical composition of the *A. pilosa* aqueous extract was determined using liquid chromatography-mass spectrometry (LC-MS). Briefly, aqueous extracts of *A. pilosa* were subjected to ultra-high performance liquid chromatography-mass spectrometry (UHPLC-MS) analysis. LC-MS was performed using a LTQ Orbitrap XL linear ion trap mass spectrometer system (Thermo Fisher Scientific Co., Waltham, MA) equipped with an electrospray ionization source. The UHPLC separations were performed with an Accela UHPLC system (Thermo Fisher Scientific) by using an Acquity BEH C18 column (1.7 μm, 100 × 2.1 mm; Waters Corp., Milford, MA). Mobile phase A was water and phase B was acetonitrile, where both phases contained 0.1% formic acid. The gradient elution, at a flow rate of 0.3 mL/min, was performed as follows: 0–1 min, 1% B (isocratic); 1–15 min, 1–30% B (linear gradient); 15–20 min, 30–60% B (linear gradient); 20–25 min, 60–100% B (linear gradient); and 25–27 min 100% B (isocratic). Full-scan mass spectra were obtained using the negative-ion mode at with an *m/z* range of 100–1000. Data-dependent tandem mass spectrometry (MSn) experiments were controlled by menu-driven software provided with the Xcalibur system (Thermo Fisher Scientific).

### ERα- and ERβ-binding assays

Estrogen receptor-binding ability was examined using an estrogen receptor (ER) competitive binding assay kit (Invitrogen, Carlsbad, CA) according to the manufacturer’s instructions. The relative affinity of the test material for ER was determined by the change in polarization value (Molecular Devices Inc., Sunnyvale, CA) in the presence of a test plant.

### Cell culture

MCF-7, an ER-positive human breast cancer cell line, was purchased from the Korean Cell Line Bank (Seoul, Korea) and cultured in RPMI-1640 containing 10% fetal bovine serum and penicillin-streptomycin solution (100 units/mL penicillin and 100 μg/mL streptomycin; Hyclone Laboratories, Inc., South Logan, UT). The cells were grown at 37°C in a humidified atmosphere of 95% air/5% CO_2_. The medium was renewed 2–3 times per week, and before reaching confluence, the cells were subcultured every 3–4 days in a 1: 4 ratio.

### Proliferation assay of MCF-7 cells (E-screen assay)

Confluent MCF-7 cells were washed twice with phosphate-buffered saline (PBS) (Hyclone Laboratories) and 0.05% trypsin-EDTA solution (Invitrogen) was added for 1 min. After trypsin-EDTA was removed, the culture was left at room temperature (~20°C) for 5–10 min; subsequently, the cells were detached, resuspended in RPMI-1640 medium, counted, and seeded into 24-well plates at a density of 2 × 10^4 ^cells/well in normal growth medium. After 24 h, the cells were completely attached to the well bottom; the medium was then aspirated and estrogen-free medium containing both phenol-red-free RPMI (Invitrogen) and 5% charcoal-dextran-stripped human serum (Hyclone Laboratories) was added. MCF-7 cells were treated with different concentrations of test material and were cultured for 144 h. In addition, test material were added to the medium at some concentrations where they showed estrogenic activity, either with or without the ER-antagonist ICI 182,780 (Tocris, Bristol, UK). 17β-Estradiol and PBS were used as the positive and negative controls, respectively.

### MTT proliferation assay

Cell proliferation was assessed after 7 d in culture, using the MTT proliferation assay. After the incubation period, cells were added with 100 μL of 5 mg/mL thiazolyl blue tetrazolium bromide (Sigma, St. Louis, MO) solution/well and were incubated further for 4 h in a humidified atmosphere (37°C in 5% CO_2_). The medium was replaced with 1 mL dimethyl sulfoxide (DMSO). The absorbance was measured at 540 nm in a microplate reader (Molecular Devices Inc., Sunnyvale, CA). Cell proliferation was expressed as percentage values compared with the negative PBS control, which was considered to represent 100% cell proliferation.

### RNA isolation

MCF-7 cells were seeded in 75-cm^2 ^culture flasks at a density of 2 × 10^4 ^cells/cm^2 ^in RPMI-1640 medium and incubated at 37°C at 5% CO_2_. On the following day, the medium was shifted to estrogen-free medium containing phenol-red-free RPMI (Invitrogen) and 5% charcoal-dextran-stripped human serum (Hyclone Laboratories) with controls or test material. After the cells were incubated for 24 h at 37°C and 5% CO_2_, they were washed twice with PBS, and total RNA was extracted from cells using the RNeasy Plus Mini Kit (Qiagen, Venlo, Netherlands), according to the manufacturer’s protocol. The quantity and purity of the total RNA obtained were determined on the basis of the absorbance at 260 and 280 nm. RNA quality was determined by gel electrophoresis on 2% agarose gels stained with ethidium bromide (0.5 μg/mL). RNA samples were stored at −20°C until use.

### Real-time RT-PCR

The expression levels of estrogen-dependent genes were determined by a real-time one-step RT-PCR performed using the SYBR Green PCR master mix (Qiagen) and a thermal cycler Rotor-Gene 3000 (Corbett Research, Mortlake, Australia), in accordance with the manufacturer’s protocol. The primer sequences for glyceraldehyde-3-phosphate dehydrogenase (GAPDH), pS2, progesterone receptor (PR), and cathepsin D are as follows (forward and reverse, respectively):

GAPDH: 5^′^-CCATGGAGAAGGCTGGGG-3^′^, 5^′^-CAAAGTTGTCATGGATGACC-3^′^; pS2: 5^′^-CATCGACGTCCCTCCAGAAGAG-3^′^, 5^′^-CTCTGGGACTAATCACCGTGCTG-3^′^; PR: 5^′^-CGCGCTACCCTGCACTC-3^′^, 5^′^-TGAATCCGGCCTCAGGTAGTT-3^′^; and cathepsin D: 5^′^-CTGAGCAGGGACCCAGATG-3^′^, 5^′^-CAGGTGGACCTGCCAGTAG-3^′^.

One step RT-PCR was performed as follows: (1) reverse transcription at 50°C for 30 min; (2) initial denaturation at 95°C for 15 min; and (3) 40 cycles of denaturation at 95°C for 30 s, annealing at 60°C for 30 s, and elongation at 72°C for 30 s. Melting-curve analysis was performed at 72–95°C to verify the specificity of amplification and was supplemented with 2% agarose gel electrophoresis of randomly selected samples. The relative quantitation values were calculated by analyzing the changes in SYBR Green fluorescence during PCR, according to the manufacturer’s instructions. C_t _values were defined as the threshold cycles at which a statistically significant increase occurred in the intensity of SYBR Green emission. Using the 2^-ΔΔCt ^method, the changes (in orders of magnitude) relative to the control were calculated. C_t _values were normalized to those for the housekeeping gene, GAPDH and the ΔC_t _values of the *A*. *pilosa*-treated cells were normalized to the mean ΔC_t _values of the controls.

### Statistical analysis

Data was expressed as mean ± SD values. Cell proliferation was expressed as percentage values compared with that for the negative PBS control, which was taken to represent 100% cell proliferation. Duncan’s multiple range tests were used to detect differences between groups when analysis of variance was significant at *p* < 0.05. Student’s *t*-test was used to establish significant differences between any 2 groups.

## Results

### HPLC analysis of *A. pilosa* aqueous extract for constituent flavonoids

**Figure 1:**
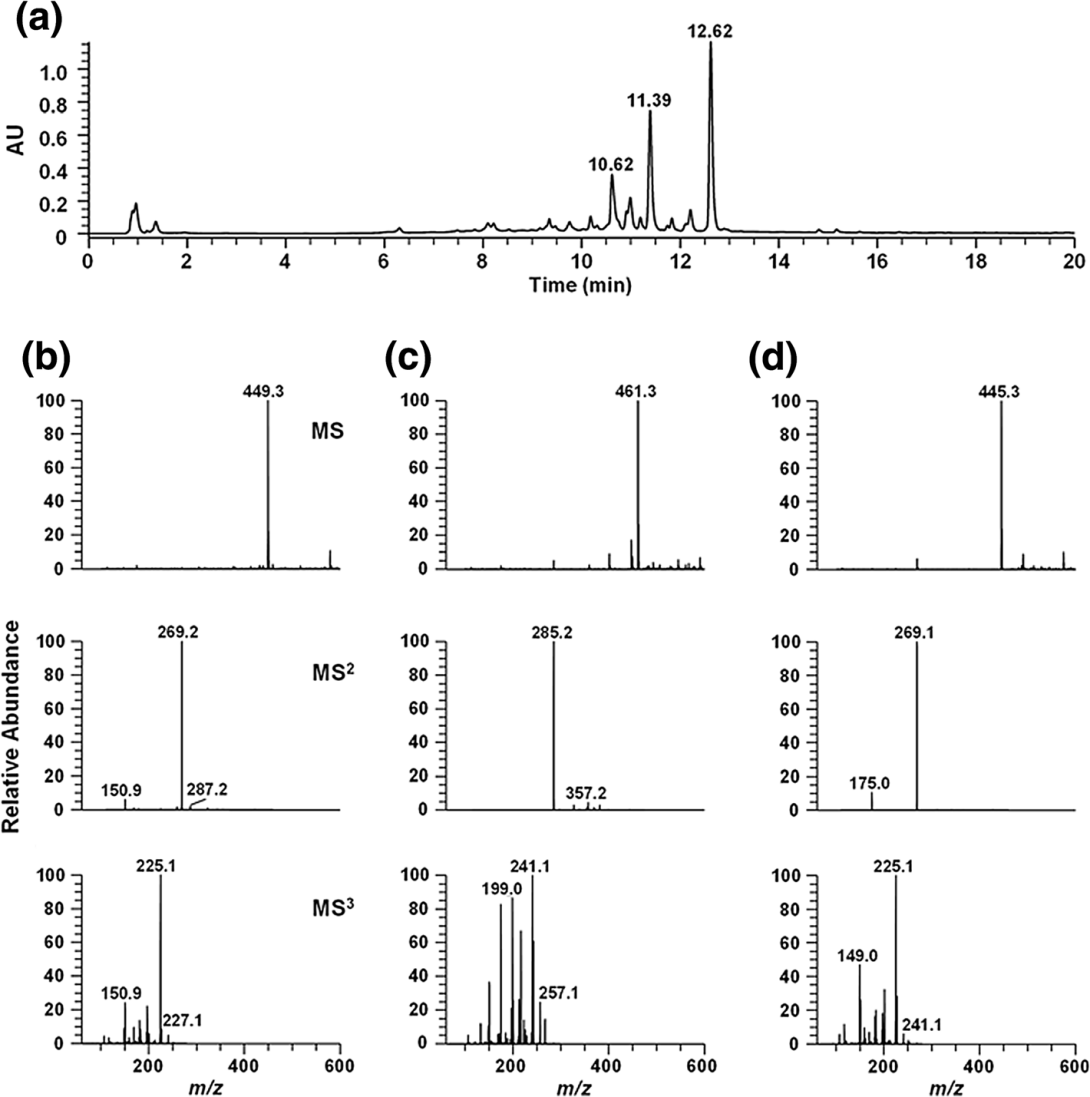
**Results of UPLC-MS analysis of the 3 main compounds of *A. pilosa *aqueous extract.** (**a**) Chromatogram at 350 nm, (**b**) mass spectrum of apigenin-hexose ([M-H]^-^, *m/z* 449.3) at an RT of 10.62 min, (**c**) luteolin-glucuronide ([M-H]^-^, *m/z* 461.3) at an RT of 11.39 min, and (**d**) apigenin-glucuronide ([M-H]^-^, *m/z* 445.3) at an RT of 12.62 min.

*A. pilosa* aqueous extract was examined for its flavonoid composition by simultaneous estimation using negative ion-mode tandem mass analysis (MS^2 ^and MS^3^). Individual constituents were identified by analysis of the MS^2 ^and MS^3 ^tandem mass spectrum (Figure 1). The 3 major peaks of the *A. pilosa* aqueous extract were identified as apigenin-hexose ([M-H]^-^, *m/z* 449.3) at an RT of 10.62 min, luteolin-glucuronide ([M-H]^-^, *m/z* 461.3) at an RT of 11.39 min, and apigenin-glucuronide ([M-H]^-^, *m/z* 445.3) at an RT of 12.62 min. The sugar moiety was defined by MS^2^ fragment ion analysis. The aglycone structure of MS^3 ^spectra was identified by an MS/MS spectrum library search [21]. The percentage content of each flavonoid was estimated with the relative peak area based on PDA (200–500 nm) data. The flavonoids present in the extract included apigenin-glucuronide (21.81%), apigenin-hexose (19.46%), and luteolin-glucuronide (13.03%).

### Competitive binding of *A. pilosa* to estrogen receptors

**Figure 2:**
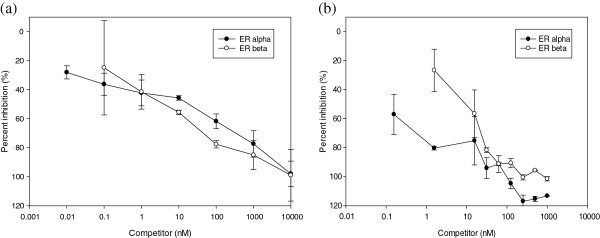
**Competitive binding curves illustrating the binding of *A. pilosa *to ERα and ERβ.** The results are expressed as mean ± SD values. (**a**) The binding affinity of E2 to ERα and ERβ, and (**b**) the binding affinity of *A. pilosa* to ERα and ERβ.

To evaluate the estrogenic activity of the aqueous extract from *A. pilosa*, the ability of *A. pilosa* to bind ERα and ERβ was assessed using a competitive binding assay based on fluorescence polarization. As shown Figure 2, this aqueous extract could bind ERs and displaced E2-bound ERα and ERβ.

### Effect of *A. pilosa* on MCF-7 cell proliferation

**Figure 3:**
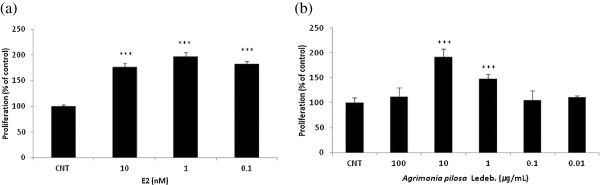
**Effect of (a) E2 (17-β estradiol) and (b) aqueous extracts of *A. pilosa *on the proliferation of MCF-7 cells.** Figures were selected as representative data from three independent experiments. Results are expressed as mean ± SD values. Statistical significance was determined using the Student’s *t* test. *** *p* < 0.001.

**Figure 4:**
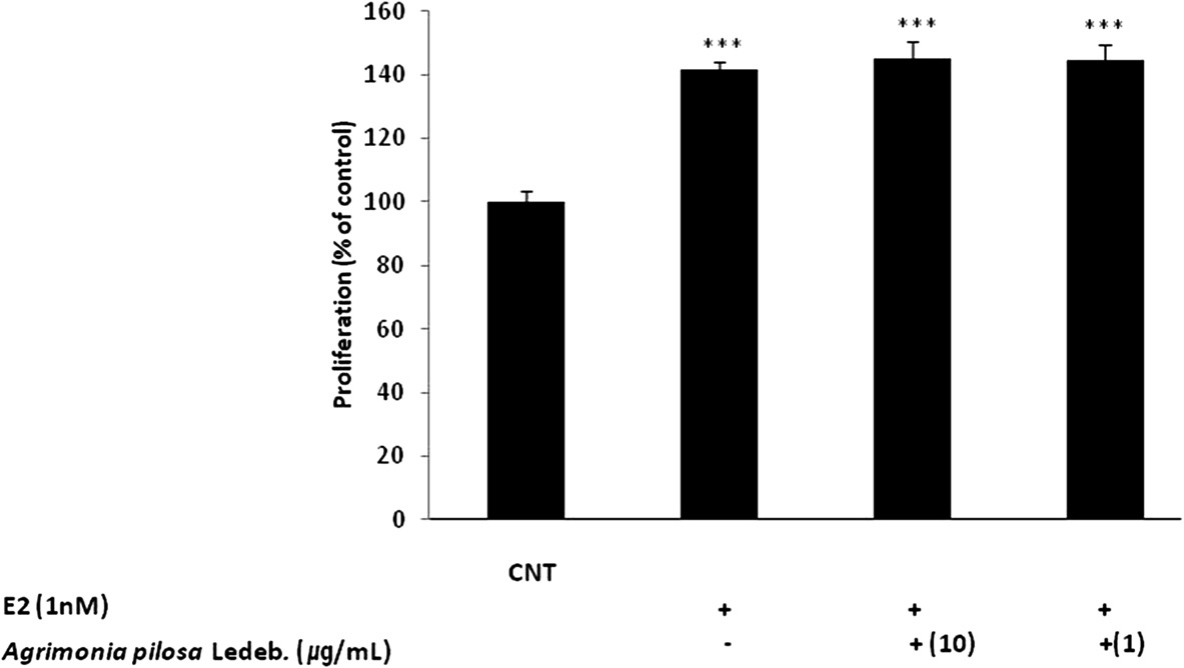
**Effect of aqueous extract of *Agrimonia pilosa *Ledeb. on E2-induced proliferation of MCF-7 cells.** Figures were selected as representative data from three independent experiments. Results are expressed as mean ± SD values. Statistical significance was determined using the Student’s *t*-test (*** *p* < 0.001).

In the E-SCREEN assay using MCF-7 cells, the proliferative effect of the aqueous extracts from *A. pilosa* was evaluated relative to that of the negative control (PBS; Figure 3). As a positive control, the estrogenic effect of 17β-estradiol on the proliferation in MCF-7 cells was measured. 17β-Estradiol caused significant proliferation in MCF-7 cells at concentrations of 10^-10^–10^-8^ M without cytotoxic activity (*p* < 0.001), and the highest proliferative effect was observed at 10^-9 ^M. *A. pilosa* induced significant stimulation of MCF-7 cell proliferation at concentrations of 1 and 10 μg/mL (*p* < 0.001). When *A. pilosa* extracts were co-treated with E2, *A. pilosa* did not antagonize E2 activity in MCF-7 cells (Figure 4).

### ER-dependent action of *A. pilosa* in MCF-7 cells

**Figure 5:**
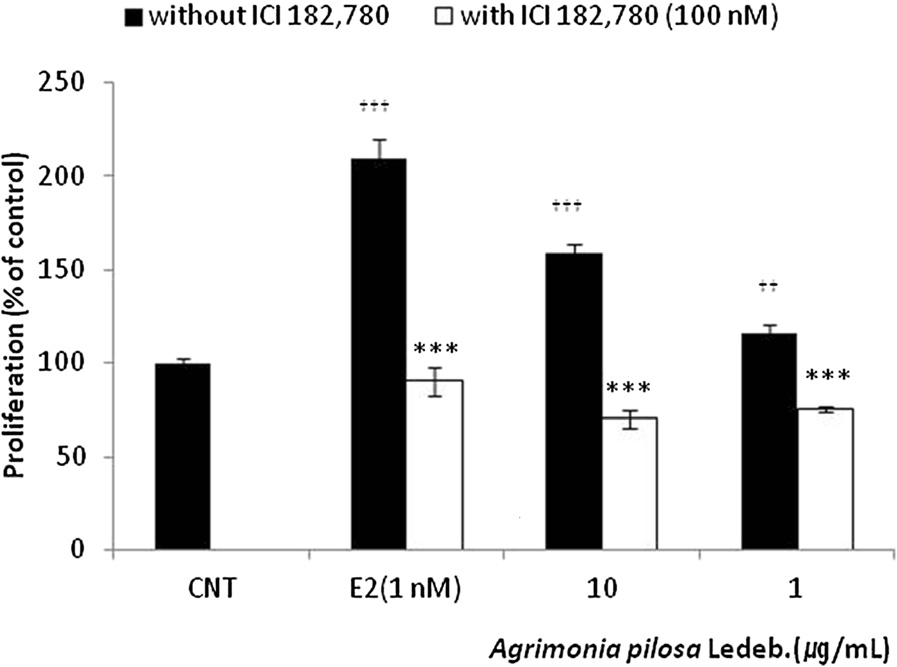
**Effect of ICI 182,780 on *Agrimonia pilosa *Ledeb.-induced proliferation in MCF-7 cells.** Figures were selected as representative data from three independent experiments. Results are expressed as mean ± SD values. Statistical significance was determined using the Student’s *t*-test. *** *p* < 0.001 (vs. without ICI 182,780), ^†††^*p* < 0.001, ^††^*p* < 0.01 (vs. control).

To investigate whether the MCF-7 cell proliferation induced by *A. pilosa* is mediated via the ER, cells were incubated with aqueous extract from *A. pilosa* in the presence or absence of the estrogen antagonist, ICI 182,780 (100 nM). The proliferation induced by *A. pilosa* and E2 was blocked by addition of ICI 182,780 (Figure 5) indicating an ER-dependent mechanism for the estrogenic effects on MCF-7 cell proliferation.

### Effect of *A. pilosa* on the mRNA levels of estrogen-dependent genes

**Figure 6:**
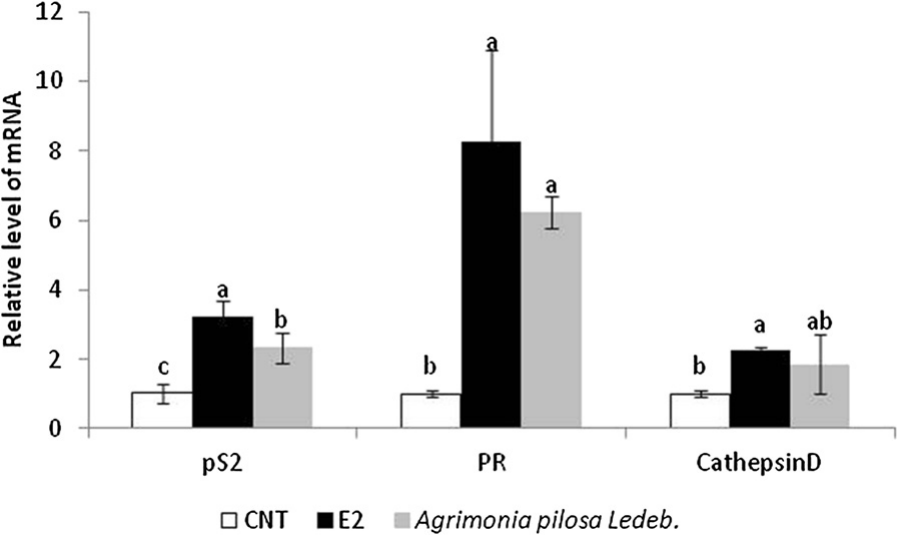
**Effect of E2 (1 nM) and *Agrimonia pilosa *Ledeb. (10 μg/mL) on the mRNA levels of estrogen-dependent genes.** Figures were selected as representative data from three independent experiments. Results are expressed as mean ± SD values. Statistical significance was determined using ANOVA followed by a Duncan’s multiple range test (*p* < 0.05).

Three estrogen-dependent genes, pS2, PR, and cathepsin D, in MCF-7 cells were selected for investigating the *A. pilosa*-induced transcriptional response through ER binding. These genes are rapidly and strongly induced by estrogens and indicate estrogenic activity [13,22,23]. Furthermore, these genes are expressed in the MCF-7 breast cancer cells used in the current study and are regulated by estrogen [24-26]. As shown in Figure 6, estrogen induced pS2 (>3-fold), PR (>8-fold), and cathepsin D (>2-fold) gene expression with respect to the control. *A. pilosa* treatment also elicited an increase in the mRNA expression of pS2, PR, and cathepsin D, resulting in 2- to 6-fold higher expression than that of the control.

## Discussion

Recent concerns have been raised regarding the side effects of the prolonged use of ERT, including an increased risk of breast and endometrial cancers. As a result, research focused on identifying natural agents with estrogen-like activity for the treatment of estrogen deficiency has increased. In light of this increased interest, dozens of edible wild plants have been screened with the goal of identifying estrogenic activity as a part of the search for new phytoestrogens. Of the plants tested, aqueous extracts from the aerial parts of *A. pilosa* have demonstrated estrogenic activity. *A. pilosa* is a traditional medicinal plant possessing anti-carcinogenic properties, anti-oxidant, and anti-inflammatory properties, and acetylcholinesterase inhibitory effects. However, little is known about the estrogenic activity of the *A. pilosa* aqueous extract.

Since the first step in the activation of ERs involves the binding of a ligand, measurement of ligand binding is important in characterizing the potential estrogenicity of test materials [27]. Therefore, the binding affinity of *A. pilosa* aqueous extract to ERs was first measured, and subsequently, *A. pilosa* was thought to bind to ERs by displacing E2 binding. The binding of estrogens or selective estrogen-receptor modulators to ERs initiates a molecular signaling cascade resulting in the transcriptional regulation of specific genes and protein synthesis [28]. Next, a proliferation assay was performed to assess the estrogen-like physiologic action of *A. pilosa* aqueous extract. Estrogens are known stimulants of cellular proliferation and the E-Screen assay measures the effects of a candidate estrogen on cell proliferation in established cell lines, such as MCF-7 [29-31]. The E-Screen assay is one of the most common reliable and valid tests for the physiologic action of estrogen. Finally, the expression of estrogen-related genes may indicate the presence of a functional estrogen signaling pathway [23,27]. This assay considers all steps of the ER-signaling pathway, including ER-ligand binding, ER expression, ER dimerization, and available co-activators. Due to the tissue specificity of endogenous ER-regulated gene (or protein) expression, the correct marker should be measured in used cell lines or tissue. In this study, 3 estrogen-dependent genes, pS2, PR, and cathepsin D, were selected to investigate the *A. pilosa*-induced transcriptional response through ER binding. These genes are expressed in MCF-7 breast cancer cells used in the present study and are regulated by estrogen, indicating the estrogenic activity of *A*. *pilosa*.

Few previous reports have investigated the chemical components of *A*. *pilosa*. Chemical studies on *A*. *pilosa* have shown the presence of polyphenols such as flavonoids [16,32]. Flavonoids and phenolic compounds are known to possess estrogenic activity [33,34]. Therefore, many studies on the estrogenic activity of natural plants attributed their estrogenicity to the presence of flavonoids and phenolic compounds [35,36]. Of the known compounds, the examples of flavonoid phytoestrogens include quercetin, apigenin, and luteolin. In the current study, the flavonoids present in the *A. pilosa* extract included apigenin-glucuronide (21.81%), apigenin-hexose (19.46%), and luteolin-glucuronide (13.03%). Further investigations to isolate and characterize the estrogenic constituents of *A. pilosa* are underway.

Phytoestrogen was found to show biphasic modulation with regard to MCF-7 cell proliferation. While genistein stimulates cell proliferation and induces the estrogen-regulated end product pS2 at low concentrations (1 pM/nM), inhibition of cell growth by ER-independent cellular mechanisms is observed at higher concentrations (>10 nM) [37]. Similarly, an abundant steroidal saponin in ginseng, ginsenoside Rg1, stimulates ER-dependent human breast cancer cell growth at low, but not high, concentrations [12]. The ethanol extract of *Ganoderma lucidum* has been reported to show both stimulation (0.1–10 μg/mL) and inhibition (>10 μg/mL) of MCF-7 cell proliferation [38]. In our study, *A. pilosa* aqueous extract also stimulated MCF-7 cell proliferation at low concentrations (1–10 μg/mL) but not at high concentrations (100 μg/mL) similar to that of other known phytoestrogens.

## Conclusions

In conclusion, to our knowledge, this study is the first to investigate the estrogenic activity of *A*. *pilosa* aqueous extract by using several *in vitro* assays in MCF-7 cells. *A*. *pilosa* aqueous extract bound to ERα and ERβ. In an MCF-7 cell proliferation assay, *A*. *pilosa* stimulated the proliferation of estrogen receptor-positive MCF-7 cells in a dose-dependent manner. Proliferation induced by E2 (1 nM) did not decrease after addition of *A*. *pilosa* aqueous extract. The proliferation induced by *A*. *pilosa* was blocked by addition of the estrogen antagonist, ICI 182,780. Furthermore, *A*. *pilosa* treatment increased the mRNA expression of estrogen-responsive genes (pS2 and PR). It was therefore concluded that the agonist effects of *A. pilosa* were primarily mediated through ER. These *in vitro* results demonstrate for the first time that *A*. *pilosa* has potent estrogenic activity and may have beneficial effects for postmenopausal women requiring ERT.

## Competing interests

The authors declare that they have no competing interests.

## Authors’ contributions

YML designed the research; YML and JHB conducted research. JBK, JSL and PSK conducted HPLC analysis. YML wrote the paper. HHJ and HRK contributed to study design and data interpretation. All authors read and approved the final manuscript.

## Pre-publication history

The pre-publication history for this paper can be accessed here:

http://www.biomedcentral.com/1472-6882/12/260/prepub
